# Antimicrobial Resistance and Molecular Epidemiology of *Staphylococcus aureus* from Hunters and Hunting Dogs

**DOI:** 10.3390/pathogens11050548

**Published:** 2022-05-06

**Authors:** Vanessa Silva, Manuela Caniça, Vera Manageiro, Madalena Vieira-Pinto, José Eduardo Pereira, Luís Maltez, Patrícia Poeta, Gilberto Igrejas

**Affiliations:** 1 Microbiology and Antibiotic Resistance Team (MicroART), Department of Veterinary Sciences, University of Trás-os-Montes and Alto Douro (UTAD), 5000-801 Vila Real, Portugal; jeduardo@utad.pt (J.E.P.); lmaltez@utad.pt (L.M.); 2 Department of Genetics and Biotechnology, University of Trás-os-Montes and Alto Douro (UTAD), 5000-801 Vila Real, Portugal; gigrejas@utad.pt; 3 Functional Genomics and Proteomics Unit, University of Trás-os-Montes and Alto Douro (UTAD), 5000-801 Vila Real, Portugal; 4 LAQV-REQUIMTE, Department of Chemistry, NOVA School of Science and Technology, Universidade Nova de Lisboa, 2829-516 Caparica, Portugal; 5 National Reference Laboratory of Antibiotic Resistances and Healthcare Associated Infections (NRL-AMR/HAI), Department of Infectious Diseases, National Institute of Health Dr. Ricardo Jorge, Av. Padre Cruz, 1649-016 Lisbon, Portugal; manuela.canica@insa.min-saude.pt (M.C.); vera.manageiro@insa.min-saude.pt (V.M.); 6 Centre for the Studies of Animal Science, Institute of Agrarian and Agri-Food Sciences and Technologies, Oporto University, 4051-401 Oporto, Portugal; 7 CECAV—Veterinary and Animal Research Centre, University of Trás-os-Montes and Alto Douro (UTAD), 5000-801 Vila Real, Portugal; mmvpinto@utad.pt; 8 Associate Laboratory for Animal and Veterinary Science (AL4AnimalS), University of Trás-os-Montes and Alto Douro (UTAD), 5000-801 Vila Real, Portugal

**Keywords:** *Staphylococcus aureus*, MRSA, transmission, dogs, human-to-dog

## Abstract

Several studies have showed that a dog-to-human transmission of *Staphylococcus aureus* occurs. Hunting dogs do not have as much contact with their owners as dogs that live in the same household as the owners; however, these dogs have contact with their owners during hunting activities as well as when hunting game; therefore, we aimed to isolate *S. aureus* from hunters and their hunting dogs to investigate a possible *S. aureus* transmission. Nose and mouth samples were collected from 30 hunters and their 78 hunting dogs for staphylococcal isolation. The species identification was performed using MALDI-TOF. The antimicrobial susceptibility profiles were accessed using the Kirby–Bauer method and respective antimicrobial resistance genes were investigated by PCR. Multilocus sequence typing (MLST) and *spa*- and *agr*-typing was performed in all *S. aureus* isolates. *S. aureus* were detected in 10 (30%) human samples and in 11 (15.4%) dog samples of which 11 and 5 were methicillin-resistant *S. aureus* (MRSA). Other staphylococci were identified, particularly, *S. pseudintermedius*. Most *S. aureus* isolates were resistant to penicillin, erythromycin, and tetracycline. Evidence of a possible transmission of *S. aureus* between human and dogs was detected in three hunters and their dogs. *S. aureus* isolates were ascribed to 10 STs and 9 *spa*-types. A moderate colonization of *S. aureus* in hunting dogs and their owners was detected in this study. A few dog-to-dog and dog-to-human possible transmissions were identified.

## 1. Introduction

Staphylococci are natural colonizers of humans and some animal species. *S. aureus* and *S. epidermidis* are the most frequent colonizers of human skin and mucous membranes [1]. Approximately 30% of the human population are asymptomatic carriers of *S. aureus* [2]. Humans colonized by *S. aureus* are at higher risk of subsequent infection, both nosocomial and community-acquired [3]. Indeed, although considered a commensal organism, *S. aureus* is an opportunist pathogen that can cause a wide range of diseases ranging from mild skin infections to severe and potentially fatal ones [4]. Methicillin-resistant *S. aureus* (MRSA) has been first described in 1961 and has become a priority pathogen causing infections increasingly difficult to treat [5]. Despite a downward trend in the prevalence of MRSA in the EU, 25% of European countries continue to have a rate of invasive isolates above 25% [6,7]. Methicillin-resistance is driven by the acquisition of the *mec* genes (*mec*A, *mec*B or *mec*C) which encodes the penicillin-binding protein 2a (PBP2a) with a low affinity for β-lactam antibiotics [8,9]. Healthcare-associated (HA) and community-associated (CA)-MRSA strains have emerged and spread widely [10]. Although other studies have reported a linkage between animal and human MRSA, it was not until 2005 that the first case of transmission between humans and animals was demonstrated [11,12]. In that study, the farmers and one pig were colonized by a MRSA strain different from those usually found in HA-MRSA and CA-MRSA [12].

The role of animals in the spread and transmission of MRSA strains in the human community is not well understood yet; however, several studies conducted with farm workers, pet owners, and veterinarians, who are at greater risk of being colonized or infected by MRSA, show epidemiological evidence that suggests MRSA transmission between human and animal hosts occur in both directions [13,14,15,16,17]. MRSA transmission between humans and their pets may be more favored due to intimate contact and sharing of the same household [18]. Furthermore, studies have reported infections in pet owners caused by methicillin-resistant staphylococci from pets and vice versa, which indicates that MRSA colonization might also represent a potential health risk for both humans and animals [19,20]. *S. aureus* is not generally considered part of the normal flora of dogs but it can be found in dogs at rates between 5% and 10% [21,22,23]. Instead, *S. pseudintermedius* predominates in dogs. It can also colonize humans at very low frequencies and usually dog owners [19]. Nevertheless, HA-MRSA and CA-MRSA lineages have been increasingly identified in dogs and cats [24,25]. Close interactions between dogs and their owners creates favorable conditions for MRSA transmission; however, unlike other dogs, hunting dogs do not live indoors with the owners, as they are primarily used in hunting activities. Nevertheless, hunting dogs have direct contact with the natural environment and with game species. Thus, in this study, we intend to analyze the possible transmission of *S. aureus* between hunting dogs and their hunting owners, as well as to verify if the clonal lineages of the isolates are related to strains frequently found in the environment and wild animals. For this, *S. aureus*, MRSA, and other methicillin-resistant staphylococci were isolated from hunting dogs and hunters, and the isolates were analyzed for their antibiotic resistance profile, virulence, and clonal lineages.

## 2. Material and Methods

### 2.1. Samples and Bacterial Isolates

From August to December 2019, a total of 108 samples were collected from 30 hunters and their 78 hunting dogs. Samples were collected using a nasal and oral swab (one swab per individual). All hunters were males, and the dogs’ ages, sex and breed were variable and are shown in Table S1. Swabs were inoculated into Brain Heart Infusion (BHI) broth containing 6.5% NaCl and incubated at 37 ℃ for 24 h. An aliquot of 100 μL was then seeded onto Baird–Parker agar and oxacillin resistance screening agar base (ORSAB) plates supplemented with oxacillin (2 mg/L) and incubated at 37 ℃ for 24–48 h. Presumptive *S. aureus* and MRSA colonies were selected and further identified. In cases where MRSA was found, samples from dogs and/or dog owners that shared the same household were screened for the presence of other methicillin-resistant staphylococci species. Confirmation of staphylococci species was performed using Matrix-Assisted Laser Desorption/Ionization Time-of-Flight (MALDI-TOF).

### 2.2. Antimicrobial Susceptibility Testing and Resistance Genes

For all staphylococci identified, susceptibility to penicillin (1 U), cefoxitin (30 μg), gentamicin (10  μg), tobramycin (10  μg), tetracycline (30  μg), chloramphenicol (30  μg), erythromycin (15  μg), clindamycin (2  μg), ciprofloxacin (5  μg), linezolid (10), and trimethoprim-sulfamethoxazole (1.25  +  23.75  μg) was examined using the Kirby–Bauer disk-diffusion method. The results were analyzed according to the EUCAST 2018 guidelines, except for kanamycin, which followed the CLSI 2017 guidelines [26,27]. The reference strain *S. aureus* ATCC 25923 was used for quality control.

According to the phenotypic resistance profile, each isolate was screened for the presence of the following antimicrobial resistance genes by PCR, as previously described [28]: *bla*Z and *mec*A (β-lactam resistance), *aac*(6’)*-aph*(2’’), *aph*(3’)-IIIa, *ant*(4’)-Ia and *str* (aminoglycosides), *erm*A*, erm*B*, erm*C*, erm*T*, msr*(A/B)*, mph*C*, lnu*A*, lnu*B*, vga*A and *vga*B (macrolides and lincosamide), *tet*K*, tet*M, *tet*L and *tet*O (tetracycline) and, *fex*A, *fex*B, *cat_pC194_*, *cat_pC221_* and *cat_pC223_* (chloramphenicol).

*S. aureus* isolates were screened for genes encoding virulence factors: hemolysins (*hla, hlb* and *hld*), exfoliative toxins (*eta* and *etb),* the leucocidin *lukS*/*F-*PV, and the toxic-shock syndrome toxin (*tst*) [29,30,31]. Additionally, the detection of the immune evasion cluster (IEC) system genes (*scn*, *chp*, *sak*, *sea* and *sep*) was also performed in *S. aureus* isolates which enabled the classification into different IEC types [32]. Finally, the presence of the virulence genes *lukS/F-*I and *siet* was investigated in all *S. pseudintermedius* isolates [33,34].

### 2.3. Molecular Typing in S. aureus Isolates

*S. aureus* isolates were typed by multilocus sequence typing (MLST) and *spa*-, *agr*- and SCC*mec*-typing. MLST was performed by amplifying and sequencing the amplicons of 7 housekeeping genes as previously described [35]. Isolates were subjected to *spa*-typing as previously described, and sequences were analyzed using Ridom Staph-Type software (version 1.5, Ridom GmbH, Wurzburg, Germany) [36]. The *agr* type of all *S. aureus* isolates was determined by the PCR as described in other studies [37]. Finally, all MRSA were characterized by SCC*mec* typing (I–V) using specific primers [38].

## 3. Results

A total of 108 samples (30 hunters and 78 hunting dogs) were analyzed in this study. From these samples, 21 (19.4%) *S. aureus* were isolated, of which 11 (52.4%) were MRSA. *S. aureus* were detected in 10 (30%) human samples and in 11 (15.4%) dog samples. Regarding the 11 MRSA isolates, 4 were isolated from humans and 7 from dogs. In cases when a hunter or a dog tested positive for *S. aureus* or MRSA, the remaining dogs and/or hunter living in the same household were screened for methicillin-resistant staphylococci. A total of 15 MRS were isolated from humans and dogs, namely, *S. pseudintermedius* (n = 5), *S. lentus* (n = 6), *S. sciuri* (n = 2), *S. cohnii*, and *S. vitulinus*. *S. lentus* were isolated from five dogs and one hunter who co-carried a MRSA strain (Table 1). *S. pseudintermedius* were isolated only from dogs, of which three co-carried MRSA and one co-carried *S. aureus*. *S. sciuri* and *S. cohnii* were isolated only from dog samples.

**Figure 1:**
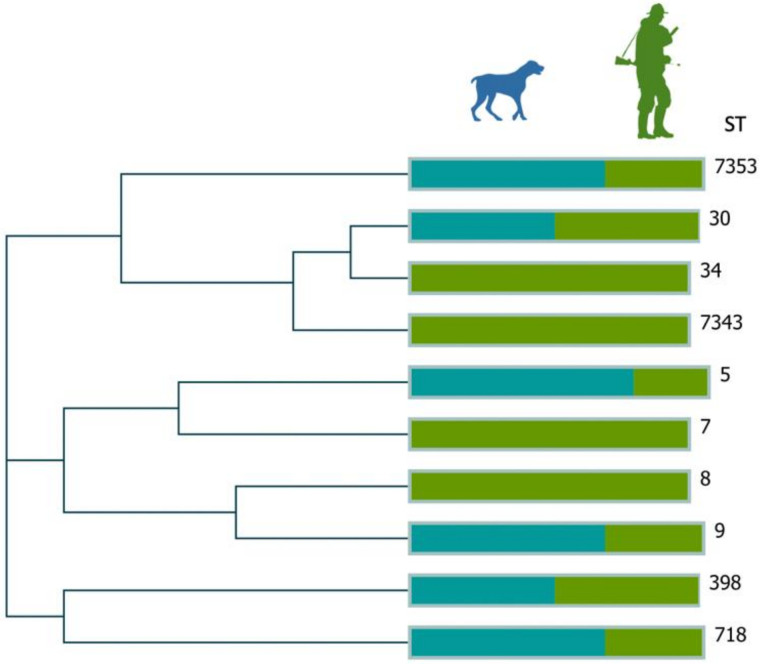
Phylogenetic tree inferred from the analysis of the MLST analysis. The tree was created using the Complete–Linkage method of Hierarchical Clustering. Hamming distance was used to measure genetic distance.

Table 1 is divided into the cases of a hunter and their respective hunting dogs. In general, MRSA isolates were ascribed to five STs (ST9, ST8, ST5, ST718, and ST7343) and five *spa*-types (t2922, t121, t11333, t012 and t179) (Figure 1). Isolates belonging to ST5, ST8, and ST718 were typed as SCC*mec* IV, whereas isolates ascribed to ST9 and ST7343 were not typeable by SCC*mec*-typing. MRSA isolates were ascribed to *agr* type II (n = 9), I and III. Only three MRSA isolates displayed a multidrug-resistant profile, all from Case 3, showing resistances to penicillin, cefoxitin, gentamycin, tobramycin, kanamycin, clindamycin, fusidic acid, and chloramphenicol encoded by the *mec*A, blaZ, *aac*(6’)-Ie-*aph*(2’’)-Ia, *aph*(3′)-IIIa, *vga*A, *lnu*B, and *cat_p221_* genes. The remaining MRSA isolates showed resistance mainly to penicillin, cefoxitin, and erythromycin conferred by the *mec*A, *bla*Z, *erm*A, and *erm*C genes. Six out of the eleven MRSA isolates harbored the *scn* and *sak* genes of the IEC system as were categorized as type E. Among the other virulence genes, *hla* (n = 9), *hlb* (n = 5), *hld* (n = 11), and *tst* (n = 2) were detected among MRSA isolates.

Methicillin-susceptible *S. aureus* (MSSA) isolates were detected in 5 hunters and 5 dogs. These isolates were ascribed to ST7353-t10042 (n = 3), ST30-t012 (n = 2), ST398-t5635 (n = 2), ST34-t166, ST718-t11333 and ST7-t091. MSSA isolates were typed as *agr* type III (n = 3), type I (n = 3) and type II (n = 1), and the ST7353-t10042 isolates were not typeable. From the ten MSSA isolates, four were susceptible to all antibiotics tested. Resistance to penicillin encoded by the *bla*Z gene was identified in five isolates and resistance to erythromycin was identified in two isolates harboring the *erm*C and *erm*T genes. MSSA isolates were grouped in IEC type E (n = 5) and G (n = 1), and one isolate carried only the *scn* gene.

As previously mentioned, in cases where MRSA was found, samples from dogs and/or dog owners that shared the same household were screened for the presence of other methicillin-resistant staphylococci species. Among the non-*aureus* staphylococci, five *S. pseudintermedius* were isolated from five dogs. All *S. pseudintermedius* isolates were resistant to penicillin and cefoxitin and carried the *mec*A gene, and therefore, they were considered MRSP. One *S. pseudintermedius* also showed resistance aminoglycosides conferred by the *aph*(2’’)-Ia, *aph*(3′)-IIIa and *str* genes. All *S. pseudintermedius* harbored the *luk*S/F-I genes and four also carried the virulence gene *siet*. All the coagulase-negative staphylococci (CoNS) isolates were recovered from dogs except for one *S. lentus*. CoNS were positive for the *mec*A gene and were resistant to penicillin. Only one CoNS (*S. lentus* VS3211) had a multidrug-resistance profile showing resistance to penicillin, gentamycin, kanamycin, clindamycin, and chloramphenicol encoded by the *mec*A, *aac*(6’)-Ie-*aph*(2’’)-Ia, *aph*(3′)-IIIa, *mph*C and *cat_p221_* genes. Moreover, this isolate was also the only CoNS to carry a virulence gene, the *hla* gene.

## 4. Discussion

*S. aureus* is part of the normal human microbiome in approximately 30% of the human population [39]. In fact, 20% of humans are permanent carriers of *S. aureus* and about 60% are intermittent carriers [39]. This data is in accordance with the frequency of *S. aureus* obtained in our study (30%) in healthy hunters. Half of the *S. aureus* isolates were MRSA, which corresponds to 16.6% of the 30 hunters tested. MRSA frequency in healthy community humans is very variable and is influenced by geographic location, demographic characteristics, sampling years, among others. Even so, several studies have reported similar frequencies to those obtained in our study. In the study by Velasco et al. none of the 550 undergraduate students carried MRSA and only 7.6% were positive for *S. aureus* [40]. Other studies have reported a frequency of MRSA in healthy humans between 14,64% and 24.7 % [22,41,42]. In our study, the frequency of *S. aureus* and MRSA in hunting dogs was 15.4% and 9%, respectively. János et al. also reported similar results with a frequency of *S. aureus* and MRSA of 11.62% and 9.30%, respectively, in kennel dogs from Romania [43]. Other studies have reported a lower prevalence of MRSA in dogs [44,45]. In contrast, in a previous study from Portugal, a higher prevalence of MRSA (30%) was detected in dogs [46]. In the only available study of *S. aureus* in hunting dogs, 36.9% and 23.7% of the dogs were nasal carriers of *S. aureus* and MRSA, respectively, which is a much higher frequency than that found in our study [47].

A few studies have reported transmission of *S. aureus* and MRSA between dogs and humans in the same household and people working in close contact with dogs [45,48,49,50,51]. Most studies rely on molecular typing techniques, such as whole genome sequencing, rep-PCR, and pulsed field gel electrophoresis, to verify dog-to-human transmission. In our study, three cases (Cases 1, 2, and 3) concerning a possible transmission between dogs and owners were identified. Our results demonstrate that hunting dogs and their owners carried *S. aureus* and MRSA strains with clonal similarity, indicating a possible transmission via direct transfer from animals to humans or vice versa. Nevertheless, it is important to point out that whole genome sequencing should have been performed to confirm the bacterial transmission. In Case 1, the hunter and his dogs share the same clonal lineage ST7353-t10042. *S. aureus* ST7353 was first reported in this study and is a single locus variant of the ST45 (CC45) with a single-point mutation in the *pta* gene. *S. aureus* belonging to CC45 is primarily known as a human-associated clone. Nevertheless, the CC45 is characterized by its diversity since it has been associated with MRSA, MSSA, HA-MRSA, CA-MRSA, commensal clones, and it has also been isolated from pets, livestock, wild animals, and the environment [52,53,54,55,56,57]. Furthermore, *S. aureus* ST45 have unique genetic differences from other *S. aureus* clades since it has been shown that ST45 branches off near the root of the *S. aureus* population [58]. *spa*-type t10042 is a rare *spa*-type and it has only been detected once since it was first reported in 2012, and is associated with human isolates in Europe [59].

A transmission of MSSA might have also occurred in Case 2 since *S. aureus* ST30-t012 was isolated from both the hunter and his hunting dog. Furthermore, the pattern of antimicrobial resistance was similar in both isolates. MSSA-ST30 is an ancestral strain of an epidemic MRSA clone which evolved into MSSA, and is also known as the Southwest Pacific clone [60]. ST30 is an international successful clone since it has been found in Australia, Europe and Asia [61]. This lineage is primarily associated with humans, but it has been also found among animals and in the environment [55,62]. Moreover, ST30-MSSA-t012 has been detected in hospitalized humans and livestock workers in Portugal, in the same region where the samples of this study were collected [63,64]. Both ST30-MSSA-t012 isolates carried the virulence gene *tst*, in addition to the hemolysins genes, which is in accordance with other studies that have shown that *S. aureus* ST30 often carries pathogenicity islands including the *tst* gene [65].

In Case 3, MRSA strains with the same clonal lineage, ST9-t2922, were isolated from the hunter and two hunting dogs. ST9 is a livestock-associated MRSA (LA-MRSA) lineage that is predominant in Asia. As in our study, this lineage lacks several important virulence genes, such as *luk*F/S-PVL. Furthermore, ST9 MRSA strains are usually multidrug resistant [66]. In our study, all ST9 isolates were resistant to penicillin, cefoxitin, aminoglycosides, clindamycin, and tetracycline encoded by the *mec*A*, bla*Z*, aac*(6’)-Ie-*aph*(2’’)-Ia, *aph*(3′)-IIIa, *lnu*B*, vga*A, *tet*K, and *tet*M genes. In addition, MRSA isolates from dogs were also resistant to chloramphenicol and harbored the *cat_p221_* gene. These minor genetic variations between the dogs and owner isolates may have evolved in the two different hosts after interspecies transmission, as was the case in other studies in which *S. aureus* was transmitted between dogs and humans [52]. MRSA *spa*-type t2922 is often associated with LA-MRSA, not so much with ST9, but rather with ST398 which is the predominant LA-MRSA lineage in Europe [67]. Even so, ST9-t2922 has been detected among pigs in China and Taiwan [68,69]. ST9-t2922 isolates were not typeable by SCC*mec* typing which may be due to the high diversity of SCC*mec* types reported among ST9 MRSA strains [66,70,71].

Dog-to-dog staphylococci transmission has been documented, particularly dogs living together in the same household [72,73]. Transmission between dogs may occur due to several factors, such as dog-to-dog contact, sharing the same water and food, and sharing the same environment. In our study, in Case 4, the hunter and three hunting dogs were colonized by MRSA strains. All dogs’ isolates belonged to ST5-t179, which suggests a possible dog-to-dog transmission. The MRSA isolates from dogs were assigned to IEC type E which indicates a possible human origin [74]; however, transmission between the hunter owner and the dogs does not seem to have occurred as the clonal lineages differ. MRSA ST5-t179 SCC*mec* type IV, also known as the Pediatric clone, is a classic human pathogen predominant in HA-MRSA, and has been repeatedly isolated from human infections in Portugal [63,75]. Nevertheless, we believe that this is the first study reporting ST5-MRSA-t179 in dogs. As for MRSA isolated from the hunter-owner, it belongs to ST8, *spa*-type t121, and SCC*mec* IV. MRSA ST8 is a common CA-MRSA clone frequently detected in the USA, which is often related with the USA300 clone; however, the presence of PVL encoding genes is a marker of the USA300 clone, and in our study, the ST8 MRSA isolate lacked this gene [76]. In fact, the epidemiology of the ST8 MRSA clone differs remarkably among world regions. For instance, in Europe, ST8 is commonly detected in community humans, but most of them are non-USA300 [76]. Nevertheless, in Case 4, a transmission of MRSP may have occurred between dogs. Two MRSP showed resistance to penicillin and cefoxitin and carried the *bla*Z and *mec*A genes; however, one MRSP isolate (VS3198) also showed resistance to aminoglycosides conferred by the *aac*(6’)-Ie-*aph*(2’’)-Ia, *aph*(3′)-IIIa, and *str* which may have been acquired after the transmission. The MSRP isolates also carried the virulence genes *luk*S/F-I and *siet*, which have been previously reported in both commensal and clinical strains, indicating that these genes may be ubiquitous in this *S. pseudintermedius* [77].

Another possible dog-to-dog transmission can be observed in Case 5. Both dogs sharing the same household carried MRSA strains belonging to ST718, t11333, and SCC*mec* IV, and they harbored the same resistance and IEC genes. *S. aureus* ST718 is a rare human-associated clone that has been reported in a few countries with regard to human infections and community humans [78,79,80,81]. Although this clonal lineage is most often associated with MSSA strains, it has also been identified among MRSA isolates that are ascribed to SCC*mec* type IV, similarly to the one obtained in this study [78]. Interestingly, in our study, one ST718-t11333 MSSA strain was also isolated from a hunter (Case 8) which shows the adaptability of this lineage. *S. aureus* ST718-t11333 has been identified among animals in only one study that was conducted with owl samples in Portugal [62]. Since hunters and their hunting dogs are in direct contact with wild game animals, transmission may also occur, especially between dogs and wild game animals.

In no other Case does the transmission of *S. aureus* between dogs and humans appear to have occurred; however, *S. aureus* was isolated from five more hunters and one dog. One hunter (Case 9) and one hunting dog (Case 6) shared the same *S. aureus* clone, ST398-t5635, but with no apparent relationship or contact between them. Animals are considered the main reservoir of *S. aureus* CC398; however, this lineage is divided into two clades: the classical LA clade and the human clade [82]. It is believed that ST398 was originally a human-associated clone, and it has adapted to animals through the loss of integrase group 3 prophages containing the IEC system genes, and with it, they acquired tetracycline resistance [83,84]; however, it has been shown that a re-adaptation of *S. aureus* CC398 to humans may occur with the acquisition of IEC [83,84,85]. Both of our CC398 isolates lacked the tetracycline resistance, which is a marker of animal adaptation; however, the human isolate was resistant to penicillin and erythromycin carrying the *bla*Z and *erm*T genes. Furthermore, this isolate also carried the *scn* gene of the IEC system. Studies have shown that CC398 related to humans and human infections often carry the *erm*T and the *chp* and *scn* genes, which may indicate a human adaptation [85,86]. It is important to point out that most MRSA and MRSP isolates did not present a multidrug-resistant profile that is common in methicillin-resistant isolates; therefore, MRSA that is simply β-lactam resistant, as many in this study are, would be of lesser concern, as it is an opportunistic pathogen, and thus, there are still a multitude of appropriate therapeutic options.

Methicillin-resistant CoNS (MRCoNS) were also isolated from both hunters and dogs in this study. A few studies have been conducted investigating the frequency of CoNS and MRCoNS in healthy dogs, and the CoNS species detected in those studies are very variable. Ma et al. reported that the most frequent CoNS among dogs in Australia was *S. sciuri*, whereas in Brazil and the United Kingdom, it was *S. epidermidis* followed by *S. simulans* and *S. epidermidis*, followed by *S. warneri*, respectively [44,87,88]. In Thailand, the most common CoNS species was *S. chromogenes* [89]. In our study, the most prevalent CoNS was *S. lentus*, which was isolated from dogs and one human. Furthermore, among all MRCoNS only one (*S. lentus* VS3211) was multidrug-resistant, and showed resistance to penicillin, aminoglycosides, clindamycin, and chloramphenicol which was conferred by the *mec*A*, aac*(6’)-Ie-*aph*(2’’)-Ia, *aph*(3′)-IIIa, *mphC*, and *cat_p221_* genes. *S. lentus* is considered to be an animal commensal and pathogen species [28]. Nevertheless, it has also been identified as the etiological agent of human infections [90]. As expected, none of the MRCoNS isolates showed phenotypic resistance to cefoxitin despite carrying the *mec*A gene. Studies have shown that the *mec*A gene may have originated from the CoNS species belonging to the *S. sciuri* group, which includes *S. sciuri*, *S. viutlinus*, and *S. lentus*, and these species often carry *mec*A homologues that do not confer phenotypic resistance [91,92]. Transmission of MRCoNS is harder to confirm since molecular typing methods are not available for all species. Nevertheless, we can hypothesize that in Case 11, the hunting dogs may be sharing the same *S. lentus* clone, since both isolates showed the same resistance pattern.

## 5. Conclusions

Genetic similarity was observed between *S*. *aureus* and MRSA isolates from hunters and their hunting dogs, suggesting possible human-to-dog and dog-to-dog transmissions which could pose a public health risk. *S. aureus* isolated from hunters and their hunting dogs living in the same household showed identical STs, *spa*-, SCCmec-, and *agr*-types, as well as similar resistance and virulence patterns. Most *S. aureus* isolates were classical human-associated clones which may point to one-way transmission from humans to dogs. Furthermore, several *S. aureus* isolates carried the genes encoding the IEC system, which reinforces a possible human origin; therefore, the role of *S. aureus* as a zoonotic pathogen is potentiated; however, although this study points to a possible *S. aureus* transmission, whole genome sequencing should be carried to confirm the human-to-dog and dog-to-dog transmissions. 

## Figures and Tables

**Table 1:** Genetic characterization of the *S. aureus*, MRSA and MRS isolates grouped by hunter and the respective dogs.

	Isolate	Host	Species	Molecular Typing				Antimicrobial Resistance		Virulence Factors	
				ST (CC)	*spa*	SCC*mec*	*agr*	Phenotype	Genotype	IEC system	Other genes
**Case 1**	VS3182	Hunter 1	*S. aureus*	7353	t10042	-	NT	PEN	*bla*Z	-	*hla, hlb, hld*
	VS3183	Dog 1	*S. aureus*	7353	t10042	-	NT	PEN	*bla*Z	E	*hla, hld*
	VS3184	Dog 2	*S. aureus*	7353	t10042	-	NT	PEN	blaZ	E	*hla, hld*
	VS3185	Dog 2	*S. pseudintermedius*	-	-	-	-	PEN, FOX	*mec*A		*luk*S/F-I, *siet*
	VS3186	Dog 3	*S. cohnii*	-	-	-	-	PEN	*mec*A		
**Case 2**	VS3187	Hunter 2	*S. aureus*	30 (30)	t012	-	III	Susceptible	-	E	*hla, hld, tst*
	VS3188	Dog 1	*S. aureus*	30 (30)	t012	-	III	Susceptible	-	-	*hla, hlb, hld, tst*
**Case 3**	VS3189	Hunter 3	***S. aureus***	9 (9)	t2922	N.T.	II	PEN, FOX, CN, TOB, KAN, CD, TET, FD	*mec*A*, blaZ, aac*(6’)-Ie-*aph*(2’’)-Ia, *aph*(3′)-IIIa, *lnuB, vga*A, *tet*M, tetK	-	*hla, hlb, hld*
	VS3190	Dog 1	***S. aureus***	9 (9)	t2922	N.T.	II	PEN, FOX, CN, TOB, KAN, CD, TET, FD, C	*mec*A, blaZ, *aac*(6’)-Ie-*aph*(2’’)-Ia, *aph*(3′)-IIIa, *vga*A, *lnuB, tet*M, tetK, *cat_p221_*	-	*hla, hlb, hld*
	VS3191	Dog 2	***S. aureus***	9 (9)	t2922	N.T.	II	PEN, FOX, CN, TOB, KAN, CD, TET, FD, C	*mec*A, blaZ, *aac*(6’)-Ie-*aph*(2’’)-Ia, *aph*(3′)-IIIa, *vga*A, *lnuB, tet*M, tetK, *cat_p221_*	-	*hla, hlb, hld*
	VS3192	Dog 5	*S. lentus*	-	-	-	-	PEN	*mec*A	-	
**Case 4**	VS3193	Hunter 4	***S. aureus***	8 (8)	t121	IV	I	PEN, FOX	*mec*A, *bla*Z,	-	*hla, hlb, hld*
	VS3194		*S. lentus*	-	-			PEN	*mec*A		
	VS3195	Dog 1	***S. aureus***	5 (5)	t179	IV	II	PEN, FOX, ERY	*mec*A, *bla*Z *erm*C,	E	*hla, hld*
	VS3196		*S. pseudintermedius*	-	-	-	-	PEN, FOX	*mec*A*, bla*Z	-	*luk*S/F-I
	VS3197	Dog 2	***S. aureus***	5 (5)	t179	IV	II	PEN, FOX, ERY	*mec*A, *bla*Z, *erm*C,	E	*hla, hld*
	VS3198		*S. pseudintermedius*	-	-	-	-	PEN, FOX, CN, TOB, KAN	*mec*A*, blaZ, aac*(6’)-Ie-*aph*(2’’)-Ia, *aph*(3′)-IIIa, *str*	-	*luk*S/F-I, *siet*
	VS3199	Dog 3	***S. aureus***	5 (5)	t179	IV	II	PEN, FOX, ERY	*mec*A*, bla*Z	E	*hla, hld*
	VS3200		*S. pseudintermedius*	-	-	-	-	PEN, FOX	*mec*A*, bla*Z	-	*luk*S/F-I, *siet*
**Case 5**	VS3201	Dog 2	***S. aureus***	718	t11333	IV	II	PEN, FOX, ERY, CD	*mec*A, *bla*Z, ermC,	E	*hld*
	VS3202	Dog 3	***S. aureus***	718	t11333	IV	II	PEN, FOX, ERY	*mec*A, *bla*Z, ermC,	E	*hld*
**Case 6**	VS3203	Dog1	*S. aureus*	398 (398)	t5635	-	I	Susceptible	-	-	*hla, hld*
	VS3204	Dog 3	*S. sciuri*	-	-	-	-	PEN	*mec*A, *bla*Z	-	
**Case 7**	VS3205	Hunter 5	*S. aureus*	34 (30)	t166	-	III	PEN, ERY	*blaZ*, *erm*C	E	*hld*
	VS3206	Dog1	*S. pseudintermedius*	-	-	-	-	PEN, FOX	*mec*A, *bla*Z	-	lukS/F-I, *siet*
	VS3207	Dog2	*S. lentus*	-	-	-	-	PEN	*mec*A	-	
**Case 8**	VS3208	Hunter 6	*S. aureus*	718	t11333		II	Susceptible	-	E	*hla, hld*
	VS3209	Dog 1	*S. vitulinus*	-	-			PEN	*mec*A		
**Case 9**	VS3210	Hunter 7	*S. aureus*	398 (398)	t5635	-	I	PEN, ERY	*bla*Z, *erm*T	*scn*	*hla, hld*
	VS3211	Dog1	*S. lentus*	-	-	-		PEN, CN, KAN, CD, C	*mec*A*, aac*(6’)-Ie-*aph*(2’’)-Ia, *aph*(3′)-IIIa, *mph*C, *cat_p221_*	-	*hla*
**Case 10**	VS3212	Hunter 8	***S. aureus***	7343	t012	N.T.	III	PEN, FOX	*mec*A, *bla*Z	-	*hla, hlb, hld*
	VS3213	Dog	*S. sciuri*	-	-			PEN	*mec*A		
**Case 11**	VS3214	Hunter 9	***S. aureus***	5 (5)	t179	IV	II	PEN, FOX, ERY,	*mec*A, *bla*Z, *erm*A	E	*hla, hld*
	VS3215	Dog 1	*S. lentus*	-	-	-	-	PEN	*mec*A, *bla*Z	-	
	VS3216	Dog 2	*S. lentus*	-	-	-	-	PEN	*mec*A, *bla*Z,	-	
**Case 12**	VS3217	Hunter 10	*S. aureus*	7	t091	-	I	PEN	*bla*Z	G	*hla, hld*

Abbreviation: NT: not typeable; PEN: penicillin; FOX: cefoxitin; CN: gentamycin; TOB: tobramycin; KAN: kanamycin; ERY: erythromycin; CD: clindamycin; TET: tetracycline; FD: fusidic acid; C: chloramphenicol; IEC: immune evasion cluster; ST: sequence type; CC: clonal complex. MRSA isolates are presented in bold.

## Data Availability

Not applicable.

## Supplementary Materials

The following supporting information can be downloaded at: https://www.mdpi.com/article/10.3390/pathogens11050548/s1, Table S1: Breed, gender, and age of all dogs sampled in this study.
