# Supplementary material for: Antimicrobial Resistance and Molecular Epidemiology of Staphylococcus aureus from Hunters and Hunting Dogs
Source: Pathogens. 2022 May 6;11(5):548. doi: 10.3390/pathogens11050548 (PMC9143024; doi:10.3390/pathogens11050548)
Supplement: Supplementary file 1 [file pathogens-11-00548-s001.zip › pathogens-1677217-supplementary.pdf]

Staphylococcus aureus from hunters and hunting dogs: molecular epidemiological evidence supporting human-to-dog and dog-to-dog transmission

Vanessa Silva, Manuela Caniça, Vera Manageiro, Madalena Vieira-Pinto, José Eduardo Pereira, Luís Maltez, Patrícia Poeta, Gilberto Igrejas

Table S1. Breed, gender, and age of all dogs sampled in this study.

Hunter	Dogs	Breed	Gender	Age
Hunter 1	Dog 1	Podengo	F	2
	Dog 2	Podengo	M	1
	Dog 3	Podengo	M	1
	Dog 4	Podengo	M	6
	Dog 5	Podengo	M	4
Hunter 2	Dog 1	Podengo	M	11
	Dog 2	Podengo	M	3
	Dog 3	Podengo	M	3
Hunter 3	Dog 1	Pointer	F	1
	Dog 2	Podengo	F	1
	Dog 3	Podengo	F	3
	Dog 4	French Spaniel	M	1
	Dog 5	Podengo	F	3
Hunter 4	Dog 1	Crossbreed	M	2
	Dog 2	Crossbreed	M	6
	Dog 3	Podengo	M	3
Hunter 5	Dog 1	Crossbreed	F	5
	Dog 2	Podengo	F	8
	Dog 3	Podengo	F	5
Hunter 6	Dog 1	Épagneul Breton	F	6
Hunter 7	Dog 1	Welsh Corgi	M	6
Hunter 8	Dog 1	Podengo	M	6
	Dog 2	Podengo	M	1
	Dog 3	Podengo	F	5
Hunter 9	Dog 1	Podengo	M	3
	Dog 2	Podengo	M	3
	Dog 3	Podengo	M	7
	Dog 4	Podengo	F	1
	Dog 5	Épagneul Breton	M	1
	Dog 6	Épagneul Breton	F	4
Hunter 10	Dog 1	Setter	M	2
Hunter 11	Dog 1	Podengo	F	4
	Dog 2	Podengo	F	9

	Dog 3	Podengo	F	4
Hunter 12	Dog 1	Grand Bleu de Gascogne	M	9
	Dog 2	Grand Bleu de Gascogne	M	6
	Dog 3	Bayrischer gebirgsschweisshund	M	2
	Dog 4	Grand Bleu de Gascogne	M	3
	Dog 5	Grand Bleu de Gascogne	M	6
	Dog 6	Grand Bleu de Gascogne	F	2
	Dog 7	Grand Bleu de Gascogne	F	6
	Dog 8	Podengo	F	3
	Dog 9	Podengo	F	7
	Dog 10	Podengo	F	1
	Dog 11	Podengo	M	1
	Dog 12	Podengo	F	4
	Dog 13	Podengo	M	7
	Dog 14	Podengo	F	3
	Dog 15	Setter	F	5
	Dog 16	Podengo	M	3
	Dog 17	Podengo	M	7
	Dog 18	Podengo	M	2
	Dog 19	Setter	M	2
Hunter 13	Dog 1	Crossbreed		0.5
	Dog 2	Podengo		4
	Dog 3	Podengo		4
	Dog 4	Pointer		3
Hunter 14	Dog 1	Podengo	F	5
	Dog 2	Podengo	F	7
Hunter 15	Dog 1	Podengo	M	10
	Dog 2	Podengo	M	4
Hunter 16	Dog 1	Pointer	F	0.5
	Dog 2	Pointer	F	0.5

	Dog 3	Setter	F	4
Hunter 17	Dog 1	Pointer	F	2
Hunter 18	Dog 1	Épagneul Breton	F	3
Hunter 19	Dog 1	Pointer	F	2
Hunter 20	Dog 1	Podengo	F	4
Hunter 21	Dog 1	Podengo	F	6
Hunter 22	Dog 1	Crossbreed	F	3
Hunter 23	Dog 1	Épagneul Breton	M	2
Hunter 24	Dog 1	Podengo	M	7
Hunter 25	Dog 1	Grand Bleu de Gascogne	F	3
Hunter 26	Dog 1	Podengo	M	0.5
Hunter 27	Dog 1	Pointer	F	8
Hunter 28	Dog 1	Setter	M	5
Hunter 29	Dog 1	Podengo	M	3
Hunter 30	Dog 1	Crossbreed	M	2
